# Why Hungarians Have Sex: Development and Validation of a Brief 15-Item Instrument (YSEX?-15H)

**DOI:** 10.1007/s10508-022-02380-x

**Published:** 2022-08-08

**Authors:** Norbert Meskó, András N. Zsidó, Béla Birkás, Cindy M. Meston, David M. Buss

**Affiliations:** 1 Institute of Psychology, University of Pécs, Ifjúság utca 6., Pécs, 7624 EU Hungary; 2 Department of Behavioural Sciences, University of Pécs, Pécs, EU Hungary; 3 Department of Psychology, University of Texas at Austin, Austin, TX USA

**Keywords:** Sexual motivation, Gender differences, YSEX?, Sexual intercourse

## Abstract

The present study developed a brief version of the Hungarian Why Sex? questionnaire (Meskó et al., 2022). The study was in part based on previously reported data obtained from several samples (*N* = 6193; 1976 men, 4217 women). Using Mokken Scaling Procedure, Item Response Model and redundancy analysis indicated that retaining three summary scales comprising five items each was the optimal solution for the brief version. The validity of the brief scale was tested with the Sexual System Functioning Scale (SSFS), the Experiences in Close Relationships Scale–Short Form (ECR-S) and, the Hungarian version of the Attachment Style Questionnaire (ASQ-H; *n* = 297, 127 men, 170 women). In addition, correlations between the long and brief versions of YSEX? with sociosexual orientation (SOI-R) and the five-factor personality construct (BFI-S) were compared (*n* = 1024, 578 women, 446 men). The results suggest that the three summary scales of the Hungarian 15-item Form of the Why Sex Questionnaire (YSEX?-15H) provide reliable and valid measures of the previously affirmed three broad sexual motives (Personal Goal Attainment, Relational Reasons, Sex as Coping). The Relational Reasons summary scale was associated with secure emotional and sexual attachment. The Personal Goal Attainment and Sex as Coping summary scales showed coherent patterns of associations with the emotional and sexual aspects of secondary attachment strategies (over- and under-functioning). The YSEX?-15H offers both researchers and practitioners a concise and useful instrument for the assessment of sexual motivation.

**Supplementary Information:**

The online version contains supplementary material available at 10.1007/s10508-022-02380-x.

## Introduction

Sexual motivation is partly captured by the reported reasons people give to engage in sexual activity. These, in turn, have an impact on the dynamics of intimate partner relationships and partners’ mental health (e.g., Davis et al., 2004; Hatfield et al., 2010; Impett et al., 2005; Meston & Buss, 2007). Extensive research suggests that one’s personal style of caring for others and sexual motives are affected by individual differences in attachment history (see Feeney & Noller, 2004 for a review). Sexual intercourse can satisfy an individual’s need for closeness, intimacy and attachment for relationship stability and reassurance (Péloquin et al., 2013). Sexual activity is influenced by a number of biopsychosocial factors, as well as contextual factors of the relationship, such as the type and duration of the relationship and the attachment style of the partners (Meston & Stanton, 2017). Attachment strategies develop early in life, which predict desired levels of intimacy and interdependence in a romantic relationship and also the sexual system in regulating sexual interactions between romantic partners (see Mikulincer & Shaver, 2012 for an overview). The person’s attachment and sexual behavioral system are thought to interact after puberty, as sex may serve attachment-related needs (e.g., intimacy, emotional closeness; Davis et al., 2004; Dewitte, 2012). Hyperactivation of the sexual system triggers intense sexual impulses and sometimes elevated anxiety in intimate settings; sexual deactivation leads to an inhibition of sexual inclinations (Birnbaum et al., 2014). For example, when experiencing closeness or intimacy, individuals with an avoidant attachment style show elevated discomfort, which may compromise their sexual and intimate experiences leading to increased dissatisfaction of one’s sex life. Another form of insecure attachment—anxious attachment—is associated with enhanced worries and fears of disapproval or rejection in the relationship. These fears can increase anxiety in the relationship and reduce sexual satisfaction and overall quality of the relationship (Dewitte, 2012). Individuals with insecure attachment styles express high needs for security in the relationship, attention and approval of the partner and express more self-doubts regarding sexuality, the main source of their conflicts in sexual encounters (Davis et al., 2004; Shaver & Mikulincer, 2006). Differences in attachment styles also result in differences in sexual motives. Individuals with more pronounced attachment anxiety show increased sexual motivation in order to reduce insecurity and establish intense closeness, whereas individuals with elevated attachment avoidance are more motivated to have sex to impress their peers or other social reference group, especially if they have the opportunity to engage in casual, uncommitted sex (Schachner & Shaver, 2004).

Sexual behaviors and motives also vary by the age of the person. For example, older couples are more emotionally and interpersonally oriented in sex compared to younger couples (Gewirtz-Meydan & Ayalon, 2019). Moreover, older individuals tend to get aroused rather more by eroticism in sex compared to younger people (Purnine & Carey, 1998), value sexual motives such as dominance, experimentation or defiance against social norm less (Browning, 2004; Wyverkens et al., 2018) and are generally less motivated to engage in sexual behaviors than younger individuals (Gray et al., 2019). Older individuals generally have lower sexual motivation than younger ones.

Several previous studies pointed out gender differences in sexual motivation (e.g., Meskó et al., 2022; Meston & Buss, 2007; Meston et al., 2009, 2020), showing that women more than men are motivated by relationship-focused reasons (e.g., I was in love; I wanted to make my partner happy; Out of mutual appeal). Men more than women are more likely to report self-focused reasons (e.g., To seek pleasure; I love sex; I wanted to be/feel happy). Compared to men, women respectively assigned more importance to emotional than physical motives for sexual activity (Denney et al., 1984; Leigh, 1989; Meston & Buss, 2007; Wyverkens et al., 2018). Klusmann (2002) reported that while both women’s and men’s intimate partners’ sexual activity and satisfaction decreased with time duration of the relationship, sexual desire decreases in women, whereas the motivation for sexual intimacy fades largely in men. Buss (2006) suggests that women show higher preference for sexual intimacy and a committed sexual relationship than men because these signs of love provide cues to the man as a partner’s long-term commitment. Men on average are more motivated by sex without necessarily having an emotional connection with the partner (Meston & Buss, 2007). Men also report more frequent sexual activity and higher sexual motivation than women (Gray et al., 2019). Cruz and Humeau (2019) found that the participants’ most important sexual motives were consistent with those found in most studied cultures, such as strengthening the relationship and increasing the partner’s well-being and satisfaction.

In a series of studies conducted among university students, Meston and Buss (2007) identified a wide array of 237 reasons for having sex. Of these, 142 reasons composed four summary scales including Physical Reasons, Goal Attainment Reasons, Emotional Reasons and Insecurity Reasons, which were consistent across genders. A principal component analysis of the items of each summary scale specified a total of 13 subscales. The Meston and Buss instrument has now been translated into several languages. This has opened the way for cross-cultural comparisons (see Gouvernet et al., 2017; Ozcan et al., 2017), but is limited in that translated adaptations of existing measures do not take into consideration the importance of cultural factors on the diversity of sexual motives. Chadwick et al. (2017) argue that different language use and ways of thinking in different cultures and subcultures can result in different ways of articulating otherwise general psychological phenomena such as sexual motivation. Using the same methodology as Meston and Buss (2007), Meskó et al. (2022) recently developed a short form Hungarian version of the YSEX? questionnaire (YSEX?-HSF). The most commonly endorsed reasons for having sex were similar among the American and Hungarian samples. While the original YSEX? comprises four summary scales and 13 subscales, the YSEX?-HSF has three summary scales and 24 subscales. These findings reflect both the cross-cultural universality and diversity of sexual motivation.

This short form version of the scale (YSEX-HSF) consists of 73 items which, while considerably shorter than the original 237-item YSEX? questionnaire, still limits its usability among researchers conducting wide-scale research. Compared to their longer versions, brief questionnaires might be more preferable for various reasons: scales including more items may provide more information and more comprehensive data but are prone to increased respondent fatigue, higher response error rates and lower rates of completion (Rolstad et al., 2011; Saucier, 1994). Moreover, psychometric quality is thought to be less affected by the shortening of scales, because of redundancy reduction; consequently, more concise self-report instruments may show even greater validity indexes (Burisch, 1997). Therefore, developing short and multidimensional questionnaires with high psychometrical quality is beneficial for both researchers and participants (Jonason & Webster, 2010). Accordingly, since previous studies employing the YSEX?-HSF confined their focus to the three summary scales reflecting on the three overarching sexual motives found among Hungarians (e.g., Birkás et al., 2020; Láng et al., 2021; Meskó et al., 2021), we aimed to develop a brief scale for assessing these three broad motives. Including a reliable and validated reduced-item version of YSEX? in surveys or field studies provides limited, but relevant data on participant’s sexual motives. The brief version can also be fruitfully utilized in qualitative research or in applied settings, where a compact assessment of sexual motives is sufficient. Developing a reliable and valid brief measure also requires establishing the nomological network (Cronbach & Meehl, 1955) by identifying links to other key psychological variables such as age, gender, attachment style and sociosexual orientation.

In line with the above mentioned findings and former studies indicating significant sex and age differences on certain measures of the 73-item YSEX?-HSF (Meskó et al., 2022), possible sex and age differences on the three summary scales of the YSEX?-15H were explored. Specifically, we expected to replicate previous findings (e.g., with the 73-item YSEX?-HSF), where men scored higher than women on Personal Goal Attainment, while women scored higher than men on Sex as Coping (no gender difference was found for Relational Reasons). Furthermore, younger participants were more likely than older ones to be sexually motivated by Relational Reasons, whereas older participants scored higher than younger ones on Personal Goal Attainment.

One explanation for individual differences in the reasons for having sex relates to the five-factor personality model (e.g., Heaven et al., 2000; Strus & Cieciuch, 2017). Previous studies linked Big Five personality traits to various components of sexuality including sexual orientation, attitudes toward sex, sexual satisfaction, sexual activity and behavior (see Allen & Walter, 2018 for an overview). Researchers also found significant associations between sexual motivation and the five-factor personality model (GSOEP Big Five Inventory, BFI-S; Hahn et al., 2012) for external validation of YSEX?-HSF (Meskó et al., 2022). Overall, the summary scales and subscales measuring sexual motivation linked positively with Extraversion, Neuroticism and Openness, whereas they correlated negatively with Agreeableness and Conscientiousness. Extraversion had the highest total number of significant associations with sexual reasons and was positively connected with all three summary scales of the YSEX?-HSF (Personal Goal Attainment, Relational Reasons and Sex as Coping). Only one scale in the sample of women revealed a significant (positive) connection with neuroticism: those reporting higher degrees of emotional instability scored higher on the Sex as Coping. Only one scale had a significant (negative) correlation with Agreeableness: those with higher levels of Agreeableness scored higher on the Personal Goal Attainment. In the men’s subsample, Conscientiousness was also related (negatively) with only one scale: those who considered themselves to be more Conscientious scored lower on the Personal Goal Attainment scale. All three YSEX?-HSF summary scores were positively associated with Openness.

Another important factor for explaining gender differences in sexual motivation is sociosexual orientation (e.g., Lippa, 2009; Schmitt, 2005). Sociosexual orientation refers to the level of restrictions individuals set on sexual relationships thus, the importance of commitment, intimacy and feelings to participate in sexual activities (Simpson & Gangestad, 1991). Similarly to personality traits, sexual strategies (Revised Sociosexual Orientation Inventory (SOI-R; Penke & Asendorpf, 2008) were also found to be relevant for external validation of YSEX?-HSF (Meskó et al., 2022). For each gender group, the connections between the global sociosexual orientation index and various sexual reasons were investigated. The same general pattern was obtained for both men and women (positive correlations were found in all cases), but while almost all summary scales and subscales of the YSEX?-HSF showed a marked association with sociosexuality in the sample of men, the same held true for only one subfactor of the Sex as Coping factor in the sample of women. In both gender groups, sociosexuality had the sole negative correlation with the Intimacy subscale.

### Research Aim

The aims of the present study were threefold: First, to reduce the number of items of the YSEX?-HSF in order to develop a shorter, more ecological instrument, without compromising its ability to assess the three major dimensions of sexual motives captured by the extended version. Analyzing the external validity of the YSEX?-15H was also aimed to ensure the psychometric quality of the brief scale creating a more concise measure of sexual motivation for early-stage research and pilot studies requiring data on the fundamental patterns of sexual motives. The second aim of the study was to test the replicability of the sex and age differences found by former studies in a relatively large sample. Our third goal was to examine the broader nomological network and in this way externally validate the new brief questionnaire, utilizing two different methods (Cronbach & Meehl, 1955). First, we tested whether the newly developed brief instrument is associated in the expected direction to the questionnaires on relationship attachment, a key factor in sexuality. Second, we expected that the associations with big five personality traits (BFI-S) and sociosexual orientation (SOI-R) used to validate the 73-item YSEX?-HSF (Meskó et al., 2022) would show similar correlations with the short YSEX?-15H scale.

## Method

### Participants and Procedure

Data were collected from 8 different samples in which 73 items of the YSEX?-HSF were applied together with different questionnaires (see Table 1 for demographic data and list of questionnaires used). The dataset was divided into two samples: a larger one for the psychometric assessment of the YSEX?-15H and a smaller one for external validation. Thus, these two samples comprised a total of eight subsamples, all of which were involved in previous studies (Birkás et al., 2020; Láng et al., 2021; Meskó et al., 2021), although some of the datasets have not been reported in detail before. The samples utilized for validations (external validation #1 with SSFS, ECR-S, ASQ-H = Sample 1; external validation #2 with SOI-R, BFI-S = Sample 7) were subsamples of the overall dataset (Total Study Sample). For further details, see Table 1. Sampling method might affect psychometric properties of scales or self-report measures thus, data analysis should reflect on this and filter possible biases. Statistical methods utilized in this study were selected accordingly (Kleka & Soroko, 2018). All data were collected online. The survey was edited in Google Forms. The link to the survey was disseminated via Facebook and via one of the most popular and influential Hungarian internet portals, Index (https://index.hu/). All participants gave informed consent, and none of them was rewarded for participation. To control the quality and reliability of survey data, duration of completion was registered and patterns of answers for a scale were analyzed in order to exclude unrealistic (i.e., too short or too long) answering times or patterns (i.e., giving the same option for all the items within a scale). The study received ethical approval as part of a larger research project on mating strategies from the Hungarian United Ethical Review Committee for Research in Psychology (Ref. No. 2017/21). All source data are available at: https://osf.io/qbjkm/?view_only=fd6b38d0f0b0400e83b0a71e5661e64f.

**Table 1:** Demographics of the samples

	Total study sample	Sample 1	Sample 2	Sample 3
N	6193	307	465	572
Woman/man, n (%)	1976/4212 (31.9/68.1%)	134/173 (43.6/56.4%)	319/146 (68.6/31.4%)	425/147 (74/26%)
Age (women) M (SD)	27.42 (9.22)	34.63 (10.71)	28.9 (9.5)	24.51 (7.74)
Age (men) M (SD)	36.47 (13.17)	26.50 (8.87)	33.6 (12.9)	29.14 (10.04)
*Highest level of education*				
Tertiary		160 (52.1%)	63%	227 (39.9%)
Secondary		143 (46.6%)	35.3%	328 (57.3%)
Primary		4 (1.3%)	1,7%	17 (3%)
*Relationship status*				
Single		10 (3.3%)	6%	109 (19.1%)
Casual relationships		43 (14%)	161 (35%)	35 (6.1%)
Committed relationship		103 (33.5%)	152 (33%)	373 (65.2)
Married		80 (26.1%)	120 (26%)	47 8.2%)
Other forms of relationships		71 (23.1%)	–	8 (1.4%)
Measures		YSEX?-HSF, SSFS, ASQ-H, ECR-S	YSEX?-HSF, RAS-H, DCI-H, STLS-H	YSEX?-HSF, DERS, FOS
Publication	This study	This study	Meskó et al. (in press)	In preparation

	Sample 4	Sample 5	Sample 6	Sample 7	Sample 8
N	800	880	984	1024	1161
Woman/man, n (%)	439/361 (55/45%)	695/185 (79/21%)	768/216 (78/22%)	578/446 (56/44%)	820/341 (71/29%)
Age (women) M (SD)	32.47 (10.93)	24.43 (8.04)	22.62 84.54)	30.47 (8.75)	
Age (men) M (SD)	46.03 (10.13)	24.01 (5.74)	25.18 (6.48)	38.14 (11.66)	
*Highest level of education*					
Tertiary		348 (39.5%)	379 (38.5%)		
Secondary		494 (56.1%)	552 (56.1%)		
Primary		38 (4.3%)	53 85.4.%)		
*Relationship status*					
Single	120 (15%)	139 (15.8%)	218 (22.2%)	28 (2,7 %)	221 (19%)
Casual relationships	60 (7.5%)	75 (8.5%)	76 (7.7%)	133 (13 %)	112 (9.6%)
Committed relationship	176 (22%)	602 (68.5%)	616 (62.6%)	281 (27,4%)	–
Married	444 (55.5%)	57 (6.5%)	56 (5.7%)	309 (30,2%)	772 (66.5%)
Other forms of relationships	–	7 (0.8%)	18 (1.8%)	273 (26,7%)	56 (4.8%)
Measures	YSEX?-HSF, LAS-HSF, SOI-R, MVS	YSEX?-HSF, PRIS-SV, FOS	YSEX?-HSF, SD3, WSWMS, FOS	YSEX?-HSF, BFI-S, SOI-R	YSEX?-HSF
Publication	Meskó et al. (2021)	In preparation	In preparation	Meskó et al. (2022) Study 3	Meskó et al. (2022) Study 2
Sex differences in YSEX?-HSF scales	Personal goal attainment: F < M, relational reasons: F = M, sex as coping: F > M			Personal goal attainment: F < M, relational reasons: F = M, sex as coping: F > M	

*YSEX*?-*HSF* = Hungarian Short Form of the Reasons for Having Sex Questionnaire; *SSFS* = Sexual System Functioning Scale; *ASQ*-*H* = Hungarian Version of the Attachment Style Questionnaire; *ECR*-*S* = Experiences in Close Relationships Scale–Short Form; *RAS*-*H* = Hungarian version of the Relationship Assessment Scale; *DCI*-*H* = Hungarian version of the Dyadic Coping Inventory; *STLS*-*H* = Hungarian version of the Sternberg Triangular Love Scale; *DERS* = Difficulties in Emotion Regulation Scale; *FOS* = Faking Orgasm Scale in Women; *SOI*-*R* = Revised Sociosexual Orientation Inventory; *LAS*-*HSF* = Hungarian Short Form of Love Attitudes Scale; *MVS* = Mate Value Scale; *PRIS*-*SV* = Short Version of the Partner and Relationship Ideal Scales; *SD3* = Short Dark Triad; *WSWMS* = Women’s Sexual Working Models Scale; *BFI*-*S* = GSOEP Big Five Inventory

### Measures

For the external validation #1 of the YSEX?-15H questionnaire, scales measuring attachment patterns and sexual system functioning (based on attachment theory) were chosen. The inclusion of attachment instruments enables the linking of proximal causes of human sexual behavior (sexual motivations) with deep structures of behavior (different patterns of attachment need). Given that the relationship between attachment and sexual motivations is well documented (e.g., Birnbaum et al., 2014; Davis et al., 2004; Feeney & Noller, 2004; Shaver & Mikulincer, 2006), we intended to link our newly developed instrument to this body of empirical evidence and associated theory. Based on the former validation process of YSEX?-HSF (73-item version), we conducted another, separate external validation (#2) where we used the same measures (SOI-R, BFI-S) as in the original study (Meskó et al., 2022) in order to make the newly developed brief version even more comparable with the original long form. For results regarding validation #2 see Table 3.

The authors first translated the items of, and instructions for, the Sexual System Functioning Scale (SSFS) and Experiences in Close Relationships Scale–Short Form (ECR-S) into Hungarian, and the obtained Hungarian version was verified with the standard back-translation technique (Brislin, 1980). Specifically, the items and instructions were retranslated into English by an independent translator unaffiliated with the study, and the two translators then resolved minor discrepancies that emerged during the back-translation procedure.

### Reasons for Having Sex Questionnaire, Hungarian Short Form (YSEX?-HSF)

The development procedure of the YSEX?-HSF involving Hungarian participants (Meskó et al., 2022) followed that of the original American version (YSEX?; Meston & Buss, 2007), item generation, factor analysis, external validation. However, in contrast with the original YSEX? that consists of four summary scales (Meston & Buss, 2007), the 73-item YSEX?-HSF has three summary scales assessing three major types of sexual motives including Personal Goal Attainment (e.g., I wanted a new experience; It was a seduction/I was seduced), Relational Reasons (e.g., I was in love; I wanted to spiritually merge with the other person) and Sex as Coping (e.g., I wanted to decrease sadness; I wanted to save the relationship). Each item is rated on a 5-point scale offering the following options: 1 = None of my sexual experiences; 2 = Few (…); 3 = Some (…); 4 = Many (…); 5 = All of my sexual experiences. Thus, higher scores reflect higher levels on each measure of sexual motives.

### Sexual System Functioning Scale (SSFS)

The SSFS (Birnbaum et al., 2014) includes 24 items measuring individual variations in sexual system functioning. The two subscales (12 items each) measure sexual hyperactivation and sexual deactivation. The Hyperactivation subscale measures desire for and anxiety about sexual intercourse (e.g., I worry about not being “good enough” in bed). The Deactivation subscale taps a lack of interest in and discomfort with sexual contact (e.g., I find it hard to feel comfortable during sexual intercourse). Higher scores on each SSFS subscale reflect higher levels of sexual hyperactivation and sexual deactivation. Both subscales showed adequate internal consistency (McDonald’s omega = 0.83 and 0.81 for Sexual Hyperactivation and Sexual Deactivation, respectively).

### Experiences in Close Relationships Scale–Short Form (ECR-S)

The ECR-S (Wei et al., 2007) is a 12-item measure of adult attachment. The scale comprises two subscales assessing two continuous attachment dimensions. Attachment Avoidance measures avoidance of intimacy with romantic partners (e.g., I do not often worry about being abandoned); Attachment Anxiety measures anxiety evoked by the actual or imagined separation from romantic partners (e.g., I need a lot of reassurance that I am loved by my close loved ones). Higher scores reflect higher levels of Attachment Anxiety and Attachment Avoidance. Both subscales showed adequate internal consistency (McDonald’s omega = 0.81 and 0.76 for Attachment Anxiety and Attachment Avoidance, respectively).

### Hungarian Version of the Attachment Style Questionnaire (ASQ-H)

The ASQ (Feeney et al., 1994; adapted to Hungarian by Hámori et al., 2016) is a 40-item scale assessing individual patterns of distinct attachment dimensions. The scale comprises five subscales, each measuring a distinct dimension, including Relationships as Secondary (RS; e.g., Achieving things is more important than building relationships), Need for Approval (NA; e.g., It’s important to me that others like me), Discomfort with Closeness (DC; e.g., I prefer to depend on myself than on other people), Preoccupation with Relationships (PR; e.g., I find that others are reluctant to get as close as I would like) and Confidence in Relationships (CR; e.g., I feel confident that other people will be there for me when I need them). Higher scores reflect higher levels of each attachment dimension. All five subscales showed adequate internal consistency (McDonald’s omega = 0.76, 0.74, 0.73, 0.73 and 0.75 for Relationships as Secondary, Need for Approval, Discomfort with Closeness, Preoccupation With Relations and Lack of Confidence, respectively).

### Revised Sociosexual Orientation Inventory (SOI-R)

The Revised Sociosexual Orientation Inventory (SOI-R; Penke & Asendorpf, 2008; Hungarian version: Meskó et al., 2014) comprises nine items assessing one’s willingness to engage in uncommitted sexual encounters. The items compose three subscales measuring the three components of behavior, attitude and desire. Responses are given on 9-point rating scales (scale anchors vary across items). Higher scores on each subscale indicate more unrestricted sociosexuality in terms of behavior, attitude and/or desire. McDonald’s omega values for the three subscales and the overall scale were as follows: Behavior: 0.81; Attitude: 0.85; Desire: 0.88; SOI-R (overall): 0.86.

### GSOEP Big Five Inventory (BFI-S)

The GSOEP Big Five Inventory (BFI-S; Hahn et al., 2012) measures five major domains of personality: Neuroticism (N), Extraversion (E), Openness to Experience (O), Agreeableness (A) and Conscientiousness (C). BFI-S consists of 15 items, three for each dimension. The answers are given on a 7-point Likert scale ranging from 1 (does not apply to me at all) to 7 (applies to me perfectly). McDonald’s omega values for the three subscales and the overall scale were as follows: Neuroticism: 0.74; Extraversion: 0.72; Openness: 0.57; Agreeableness: 0.45; and Conscientiousness: 0.65, respectively.

### Statistical Analyses

First, we conducted a redundancy analysis using the Mokken Scaling Procedure (MSP; Mokken & Lewis, 1982), since visual inspection of the data indicated highly synonymous items. The MSP identifies items that cluster together as a result of high similarity or synonymous content. The analysis returns a scalability coefficient (H) ranging from 0 to 1 for each possible pair of items. The closer the item pair is in similarity, the higher the H value will be. We set two criteria to identify redundant item pairs: (1) An H value greater than 0.50 (Mokken, 1971) and (2) Visual verification of synonymy. In each redundant case, the item judged by the authors as having a better wording was retained, while the other item was removed.

An item response analysis (IRT) was then performed using the unidimensional Graded Response Model (GRM; Samejima, 1968) separately for each summary scale of the YSEX?-HSF. This model specifies a discrimination parameter (a) for each item, which shows how closely the item is related to the latent trait. The higher this a value is, the better discrimination property an item has. The best five items (with the highest a values) were selected from among those with high discrimination ability (a > 1.35; Baker, 2001). Utilizing this method might reduce the culture dependency of scales with including only items, which are the most discriminant regarding respondents high or low on the latent trait and provide the most information of the respondent. Item information curves (IICs) were then calculated for all items and test information functions (TIF) for all summary scales.

Next, we calculated McDonald’s ω coefficients to check the internal consistency of each summary scale of the YSEX?-HSF. We chose the McDonald’s omega over Cronbach’s alpha because it allows a more accurate measure of internal consistency when the assumptions of the tau-equivalent model (i.e., violation of the equal-item variance) is not met (Dunn et al., 2014). The correlation between the YSEX?-15H and the YSEX?-HSF was assessed after correcting for redundancy due to shared items (Levy, 1967).

The Personal Goal Attainment and Sex as Coping summary scales had a negative binomial distribution, therefore we performed a Box–Cox transformation to allow for parametric testing.^1^ After the transformation, the obtained skewness and kurtosis values ( <|1.15|) and a visual inspection of the Q–Q plots indicated close-to-normal distributions. Then, we tested for gender differences on all three summary scales with independent samples t-tests and for correlations with age using Pearson’s coefficients. Due to the large sample size, the Bayesian versions of these tests were also conducted to obtain BF10 values.

To test the psychometric properties of the YSEX?-15H, a confirmatory factor analysis (CFA) was performed on an independent dataset with the robust weighted least squares with mean and variance adjusted (WLSMV) estimator. Model fit assessment was based on the comparative fit index (CFI), the Tucker–Lewis index (TLI), the root mean square error of approximation (RMSEA) and the standardized root mean squared residual (SRMR). The cutoff values of good model fit were CFI/TFI ≥ 0.95 (Hu & Bentler, 1998) and RMSEA/SRMR ≤ 0.08 (Browne & Cudeck, 1993).

The external validity of the YSEX?-15H was tested with Pearson’s coefficients for the correlations between each summary scale and each subscale of the SSFS, ECR-S and ASQ-H. The obtained skewness and kurtosis values ( <|1.15|) and a visual inspection of the Q–Q plots indicated approximately normal distribution, which justified the use of Pearson’s coefficients.

## Results

### Redundancy Analysis

The Mokken analysis indicated 19 synonymous item pairs (involving 23 items). Of each redundant item pair, the item judged to have better face validity and clearer wording was retained. The retained item were 49, 51, 54, 55, 58, 61, 64, 69, 70 and 71. See Supplement 1 for exact H coefficients.

### Item Response Analysis

In order to obtain a brief version that would equally represent each summary scale of the YSEX?-HSF, the five items with the highest a values were retained from each scale, including 16, 17, 18, 19, 20 (Personal Goal Attainment), 27, 36, 39, 47, 48 (Relational Reasons), and 61, 64, 66, 70, 71 (Sex as Coping). Supplement 2 shows the parameter values for all items. See Supplement 3 and 4 for the final version of the YSEX?-15H with a short evaluation guide in English and in Hungarian, respectively.

**Fig. 1:**
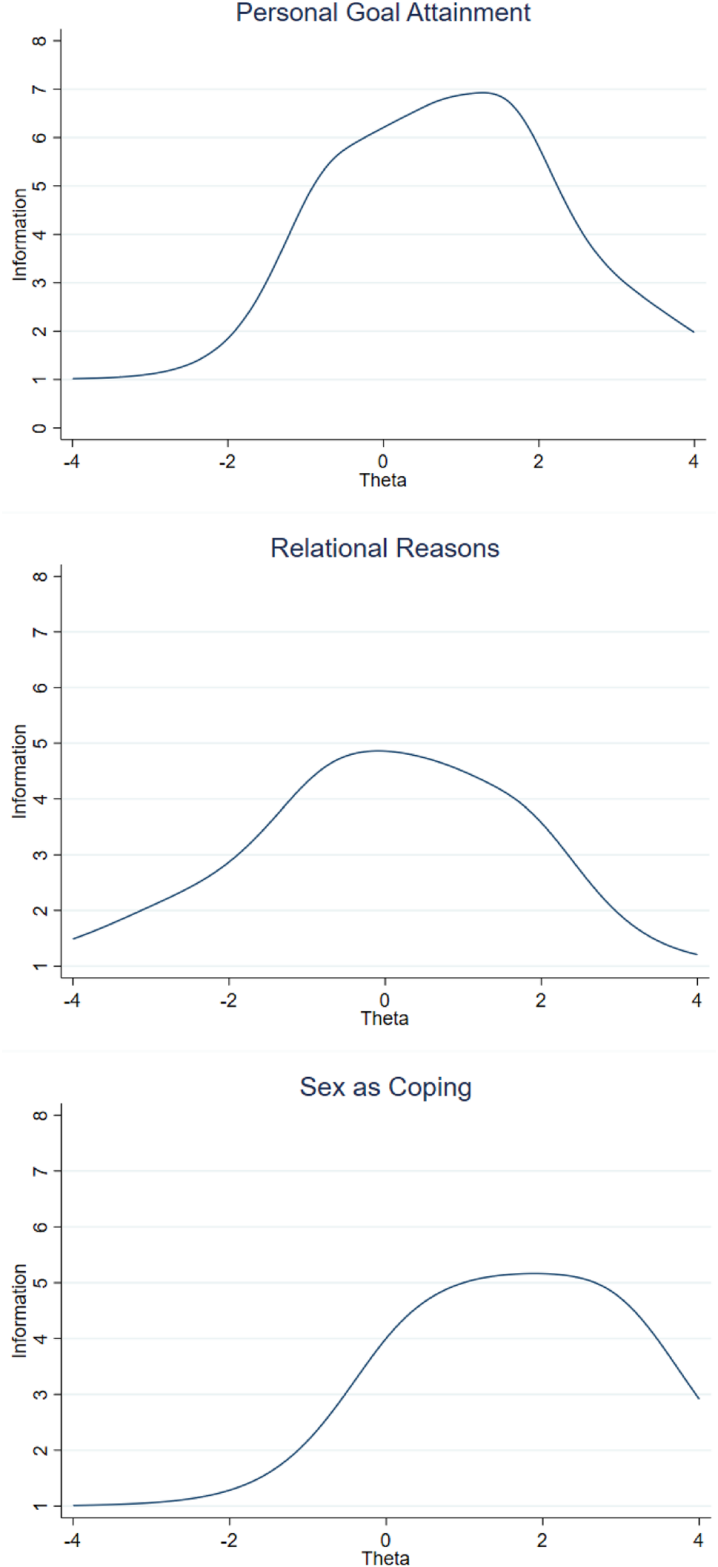
Test Information Function for the three 5-item summary scales of the YSEX?-15H

The obtained Test Information Function curves indicated that each brief scale provided a reliable measure of the respective latent variable on a wide range of values (Personal Goal Attainment on -2 to 4 SDs, Relational Reasons on − 3 to 3 SDs and Sex as Coping on − 1 to 4 SDs; see the TIF curves in Fig. 1).

### Intercorrelations Between the YSEX?-15H and the YSEX?-HSF, and Reliability of the Brief Scales

All three summary scales of the YSEX?-15H showed high positive correlations with the respective scales of the YSEX?-HSF (r = 0.81 for Personal Goal Attainment, r = 0.84 for Relational Reasons and r = 0.81 for Sex as Coping). All three brief scales showed adequate internal consistency (McDonald’s omega = 0.79, 0.75 and.73, respectively).

### Gender Differences

**Fig. 2:**
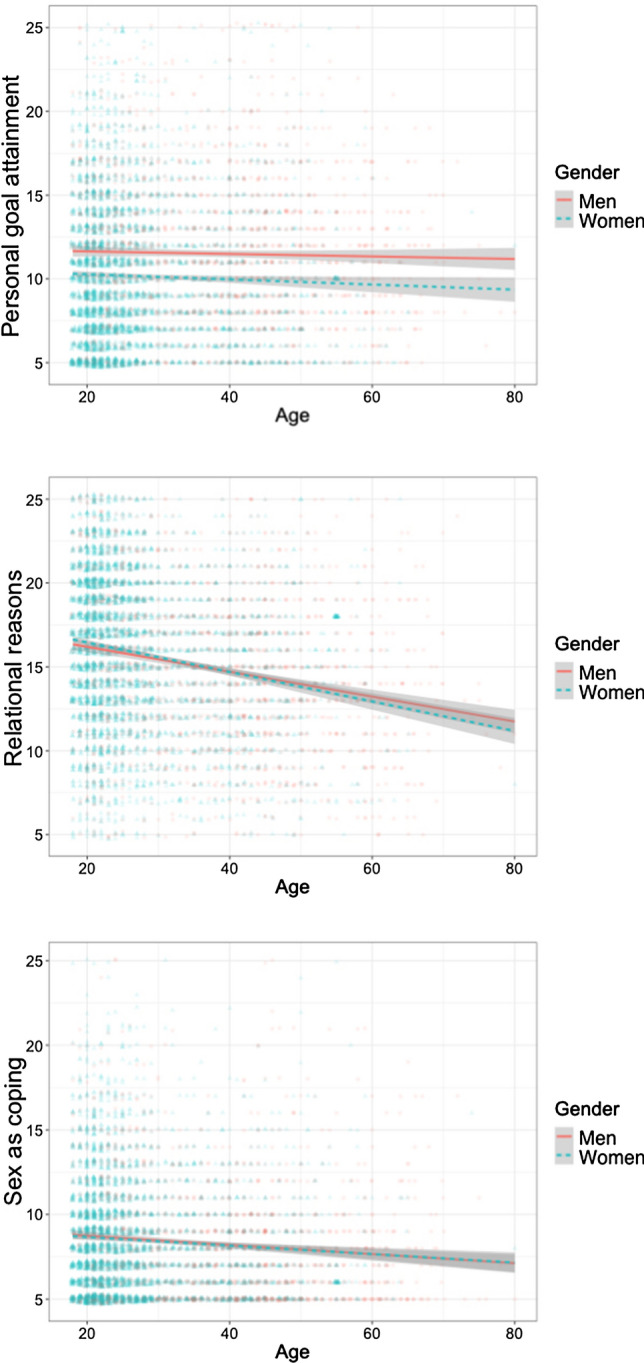
Age and gender differences on the three summary scales of the YSEX?-15H

Men scored higher than women on Personal Goal Attainment (*t*(6191) = 12.26, *p* < 0.001, Cohen’s *d* = 0.33, 95% CI = 0.28 to 0.39, BF10 = 4.43 × 1030). Women scored higher than men on Relational Reasons (*t*(6191) = 6.65, *p* < 0.001, Cohen’s *d* = 0.18, 95% CI = 0.13 to 0.24, BF10 = 1.10 × 108). Although women scored significantly higher than men on Sex as Coping, the effect size was very low, and the BF10 value indicated an equal evidence to H0 and H1 (*t*(6191) = 2.68, *p* = 0.007, Cohen’s *d* = 0.07, 95% CI = 0.02 to 0.13, BF10 = 1.11). The gender differences obtained for each summary scale of the YSEX?-15H are presented in Fig. 2.

### Age Differences

Participants’ age showed a low positive association with Personal Goal Attainment (*r* = 0.034, 95% CI = 0.009 to 0.059, *p* = 0.008, BF10 = 0.543) and low negative associations with Relational Reasons (*r* = − 0.206, 95% CI = − 0.182 to − 0.230, *p* < 0.001, BF10 = 3.28 × 1056) and Sex as Coping (*r* = − 0.090, 95% CI = − 0.065 to − 0.115, *p* < 0.001, BF10 = 1.33 × 109). The age differences obtained for each summary scale of the YSEX?-15H are presented in Fig. 2.

### Validation of the YSEX?-15H

The external validity #1 of the YSEX?-15H was tested with Pearson’s coefficients for the correlations between each summary scale and each subscale of the SSFS, ECR-S and ASQ-H. The YSEX?-15H scales showed weak to moderate correlations with the other subscales, except for ACQ CR, providing evidence of external validity (for further details, see Table 2).

**Table 2:** Pearson correlational coefficients with 95% confidence intervals (CI; U – upper, L – lower) between the YSEX?-15H summary scales and each subscale of the SSFS, ECR-S and ASQ-H questionnaires

	Scale		1	2	3	4	5	6	7	8	9	10	11	12
1	Personal Goal Attainment	r	–											
		*p*-value	–											
		95% CI U	–											
		95% CI L	–											
2	Relational Reasons	r	0.237	–										
		*p*-value	< 0.001	–										
		95% CI U	0.342	–										
		95% CI L	0.127	–										
3	Sex as Coping	r	0.448	0.378	–									
		p-value	< 0.001	< 0.001	–									
		95% CI U	0.534	0.472	–									
		95% CI L	0.352	0.276	–									
4	SSFS Hyperactivation	r	**0.294**	**0.188**	**0.341**	–								
		*p*-value	< 0.001	0.001	< 0.001	–								
		95% CI U	0.394	0.296	0.438	–								
		95% CI L	0.186	0.076	0.236	–								
5	SSFS Deactivation	r	− 0.076	− **0.213**	**0.131**	0.188	–							
		*p*-value	0.190	< 0.001	0.024	0.001	–							
		95% CI U	0.038	− 0.101	0.241	0.296	–							
		95% CI L	− 0.188	− 0.319	0.017	0.076	–							
6	ECR-S Avoidance	r	**0.321**	− **0.116**	**0.223**	0.304	0.206	–						
		*p*-value	< 0.001	0.046	< 0.001	< 0.001	< 0.001	–						
		95% CI U	0.419	− 0.002	0.328	0.404	0.312	–						
		95% CI L	0.215	− 0.227	0.112	0.197	0.094	–						
7	ECR-S Anxiety	r	**0.258**	**0.163**	**0.219**	0.546	0.145	0.189	–					
		*p*-value	< 0.001	0.005	< 0.001	< 0.001	0.012	0.001	–					
		95% CI U	0.361	0.272	0.324	0.621	0.255	0.296	–					
		95% CI L	0.148	0.050	0.108	0.461	0.032	0.077	–					
8	ASQ Relationship as Secondary	r	**0.290**	0.021	**0.237**	0.312	0.181	0.461	0.184	–				
		*p*-value	< 0.001	0.719	< 0.001	< 0.001	0.002	< 0.001	0.001	–				
		95% CI U	0.391	0.134	0.342	0.412	0.288	0.546	0.292	–				
		95% CI L	0.182	− 0.093	0.127	0.206	0.068	0.367	0.072	–				
9	ASQ Need for Approval	r	**0.141**	0.045	**0.228**	0.495	0.343	0.231	0.422	0.296	–			
		*p*-value	0.015	0.436	< 0.001	< 0.001	< 0.001	< 0.001	< 0.001	< 0.001	–			
		95% CI U	0.251	0.158	0.334	0.577	0.440	0.336	0.511	0.397	–			
		95% CI L	0.028	− 0.069	0.118	0.404	0.239	0.120	0.324	0.189	–			
10	ASQ Discomfort with Closeness	r	**0.175**	0.068	**0.285**	0.357	0.193	0.487	0.197	0.428	0.329	–		
		*p*-value	0.003	0.239	< 0.001	< 0.001	< 0.001	< 0.001	< 0.001	< 0.001	< 0.001	–		
		95% CI U	0.283	0.181	0.386	0.452	0.301	0.569	0.304	0.517	0.426	–		
		95% CI L	0.062	− 0.046	0.177	0.253	0.081	0.395	0.085	0.330	0.223	–		
11	ASQ Preoccupation with Relationships	r	**0.162**	**0.195**	**0.291**	0.510	0.237	0.066	0.638	0.217	0.589	0.185	–	
		*p*-value	0.005	< 0.001	< 0.001	< 0.001	< 0.001	0.259	< 0.001	< 0.001	< 0.001	0.001	–	
		95% CI U	0.271	0.302	0.391	0.589	0.342	0.178	0.701	0.323	0.659	0.293	–	
		95% CI L	0.049	0.083	0.183	0.420	0.127	− 0.049	0.565	0.105	0.509	0.073	–	
12	ACQ Confidence in Relationships	r	− 0.065	0.049	− 0.103	− 0.300	− 0.236	− 0.356	− 0.279	− 0.341	− 0.443	− 0.528	− 0.312	–
		*p*-value	0.266	0.403	0.076	< .001	< .001	< .001	< .001	< .001	< .001	< .001	< .001	–
		95% CI U	0.049	0.162	0.011	− 0.193	− 0.126	− 0.252	− 0.171	− 0.237	− 0.346	− 0.440	− 0.206	–
		95% CI L	− 0.177	− 0.065	− 0.214	− 0.400	− 0.341	− 0.451	− 0.381	− 0.438	− 0.530	− 0.605	− 0.411	–

*SSFS* Sexual System Functioning Scale; *ECR*-*S* Experiences in Close Relationships Scale – Short Form; *ASQ*-*H* Hungarian Version of the Attachment Style Questionnaire

The external validity #2 of the YSEX?-15H was tested using Pearson’s coefficients for the correlations between BFI-S, SOI-R and the summary scales of YSEX?-HSF and YSEX?-15H. The results show that the correlations of YSEX?-HSF and YSEX?-15H were highly similar with sociosexuality and personality (for further details, see Table 3).

**Table 3:** Pearson correlational coefficients with 95% confidence intervals (CI; U – upper, L – lower) between SOI-R, BFI-S, the summary scales of YSEX?-15H and YSEX?-HSF

		YSEX?-HSF		YSEX?-HSF		YSEX?-HSF		YSEX?-15H		YSEX?-15H		YSEX?-15H	
		Personal Goal Attainment		Relational Reasons		Sex as Coping		Personal Goal Attainment		Relational Reasons		Sex as Coping	
YSEX?-HSF Relational Reasons	r	0.51***		–									
	95% CI Upper	0.56		–									
	95% CI Lower	0.47		–									
YSEX?-HSF Sex as Coping	r	0.56***		0.54***		–							
	95% CI Upper	0.60		0.58		–							
	95% CI Lower	0.52		0.49		–							
YSEX?-15H Personal Goal Attainment	r	0.88***		0.51***		0.45***		–					
	95% CI Upper	0.89		0.55		0.50		–					
	95% CI Lower	0.87		0.46		0.40		–					
YSEX?-15H Relational Reasons	r	0.48***		0.87***		0.49***		0.47***		–			
	95% CI Upper	0.53		0.89		0.54		0.52		–			
	95% CI Lower	0.44		0.86		0.45		0.42		–			
YSEX?-15H Sex as Coping	r	0.47***		0.51***		0.87***		0.40***		0.47***		–	
	95% CI Upper	0.51		0.56		0.89		0.45		0.52		–	
	95% CI Lower	0.42		0.46		0.86		0.35		0.42		–	
SOI-R Total	r	0.53***		0.04		0.08**		0.49***		0.08**		0.03	
	95% CI Upper	0.57		0.10		0.14		0.53		0.15		0.09	
	95% CI Lower	0.48		− 0.02		0.02		0.44		0.02		− 0.03	
SOI-R Behavior	r	0.50***		0.04		0.10**		0.43***		0.07*		0.02	
	95% CI Upper	0.54		0.10		0.16		0.48		0.13		0.09	
	95% CI Lower	0.45		− 0.02		0.04		0.38		0.01		− 0.04	
SOI-R Attitude	r	0.39***		0.00		0.02		0.37***		0.07*		− 0.01	
	95% CI Upper	0.44		0.06		0.08		0.42		0.13		0.05	
	95% CI Lower	0.34		− 0.06		− 0.04		0.31		0.01		− 0.07	
SOI-R Desire	r	0.39***		0.06*		0.08**		0.37***		0.06*		0.05	
	95% CI Upper	0.44		0.12		0.14		0.42		0.12		0.12	
	95% CI Lower	0.34		0.00		0.02		0.31		0.00		− 0.01	
BFI-S Extraversion	r	0.16***		0.17***		0.08*		0.17***		0.16***		0.07*	
	95% CI Upper	0.22		0.23		0.14		0.23		0.22		0.13	
	95% CI Lower	0.10		0.11		0.02		0.11		.10		0.01	
BFI-S Neuroticism	r	0.01		0.03		0.17***		− .02		0.04		0.10***	
	95% CI Upper	0.07		0.09		0.23		0.04		0.10		0.16	
	95% CI Lower	− 0.05		− 0.03		0.11		− 0.09		− 0.02		0.04	
BFI-S Agreeableness	r	− 0.12***		0.02		− 0.06		− 0.10***		0.01		− 0.04	
	95% CI Upper	− 0.06		0.09		0.00		− 0.04		0.07		0.02	
	95% CI Lower	− 0.18		− 0.04		− 0.12		− 0.17		− 0.05		− 0.10	
BFI-S Conscientiousness	r	− 0.04		0.05		− 0.06*		− 0.00		0.05		− 0.05	
	95% CI Upper	0.02		0.12		− 0.00		0.06		0.11		0.01	
	95% CI Lower	− 0.10		− 0.01		− 0.12		− 0.06		− 0.01		− 0.11	
BFI-S Openness	r	0.12***		0.16***		0.06		0.13***		0.12***		0.03	
	95% CI Upper	0.18		0.22		0.12		0.19		0.18		0.09	
	95% CI Lower	0.06		0.10		− 0.00		0.07		0.06		− 0.03	

*p < .05, ** *p* < .01, *** *p* < .001*;* YSEX?-HSF = Reasons for Having Sex Questionnaire, Hungarian Short Form (73-item version); YSEX?-15H = Reasons for Having Sex Questionnaire, Brief Hungarian Form (15-item version); *BFI*-S Big Five Inventory Short Version; *SOI*-*R* Revised Sociosexual Orientation Inventory

## Discussion

### Development of the Brief Scale/ Item Reduction

The results indicated that all three summary scales of the YSEX?-15H highly correlated with the respective summary scales of the YSEX?-HSF. A systematic item redundancy analysis ensured that the abridgment procedure would not involve substantial information loss in terms of the three broad sexual motives. Importantly, this brief Hungarian version of YSEX? provides as much information as the YSEX?-HSF in regard to the three broad sexual motives.

The final 15-item YSEX?-15H showed high internal consistency and reliability on a large sample of Hungarian adults. The IRT analysis indicated that most items well discriminated between individuals on a range of the latent trait, while the obtained Test Information Function curves and McDonald’s omega values demonstrated the reliability of the information provided by the three summary scales of the YSEX?-15H. All subscales had a positive shift on the latent trait, which reflects the validity of this instrument and shows its usefulness in exploring possible motives in various samples. In sum, the YSEX?-15H is a psychometrically sound brief instrument that provides valid and reliable measures of the three broad sexual motives accounting for most individual differences observed among Hungarian people.

### Gender Differences

Another aim of present study was to test the replicability of the sex and age differences of YSEX?-15H in a large sample. Gender differences in sexual motivation assessed by the YSEX?-15H are only partly consistent with those previously obtained with the YSEX?-HSF (Meskó et al., 2022). Both versions indicated that men scored substantially higher than women on Personal Goal Attainment. In contrast, while women scored significantly higher on Relational Reasons as measured by the YSEX?-15H, no comparable sex difference was obtained with the YSEX?-HSF. These results partially replicate previous empirical findings on sexual motivation (e.g., Armstrong & Reissing, 2015; Hill & Preston, 1996; Whalen, 1966). Men more than women tend to prefer self-centered, unemotional reasons for their sexual encounters (e.g., Gray et al., 2019; Meskó et al., 2021, 2022; Meston & Buss, 2007), whereas women tend to prefer emotional, relationship-oriented sexual reasons that presumably enhance the intimate bond (e.g., Denney et al., 1984; Leigh, 1989; Meskó et al., 2021, 2022; Meston & Buss, 2007; Wyverkens et al., 2018). These gender differences in sexual motivation support the notion that the divergent patterns of desire for sexual intercourse is part of the different mating psychology of men and women (e.g., Conroy-Beam et al., 2019; Crosby et al., 2021; Walter et al., 2021).

In sum, men are more likely to pursue self-focused sexual goals as compared to women, who are more motivated by relationship-related reasons than men are, while men and women are equally motivated to deal with their emotional problems by engaging in sexual activity. Some studies (e.g., Cooper et al., 1998; Patrick et al., 2011) found that men reported higher coping motivation than women, while in other studies, conversely, women reported higher coping motivation than men (e.g., Meskó et al., 2021, 2022). Still other studies found no gender differences in coping motivation (e.g., Barber & Cooper, 2014; Jardin et al., 2017). These contradictory results on coping motives may suggest that both women and men use sex to cope with emotional difficulties, but for different reasons and in different social/emotional contexts.

Thus, the gender differences noted in the YSEX?-15H study seem to be theoretically well justified. It is unclear, however, why a different pattern of gender differences exists between the 15-item and 73-item versions of YSEX? One possibility is differences in methodology between studies. Namely, due to the use of the redundancy analysis (Mokken & Lewis, 1982), the number of items excluded from each scale was not the same in both studies. It is possible that differences noted in the 73-item YSEX? were significant because it contained a higher number of items referring to the same motive, only with different wording. Accordingly, not all relevant aspects or traits had equal weight in forming the total score of the scale. As such, we believe the current measure is more accurate and measures without bias because every trait or aspect is measured with one item and within the scales, these are balanced.

### Age Differences

The age differences previously obtained with the YSEX?-HSF (Meskó et al., 2022) were only partially replicated with the YSEX?-15H in the present study. Both studies found a significant positive association between Personal Goal Attainment and participants’ age. Older participants as compared to younger ones consistently assigned higher importance to self-focused reasons for engaging in sexual activity; nonetheless, the extremely low correlation obtained in the present study provided only weak evidence for this. Both studies found a significant negative correlation between Relational Reasons and age; younger participants were more sexually motivated than older ones by factors influencing developments in their intimate partner relationships. These results corroborate the findings previously obtained with the YSEX?-HSF (Meskó et al., 2022), and they are also consistent with other related findings, which show that while sexual motivation in young adulthood may serve as a means to find an intimate partner, it primarily contributes to individual partners’ quality of life at older ages when reproduction-related goals are no longer central to them (e.g., Gewirtz-Meydan & Ayalon, 2019; Gray et al., 2019; Hatfield & Rapson, 2015; Klusmann, 2002; Purnine & Carey, 1998; Wyverkens et al., 2018).

### External Validity Assessment

The tertiary aim of the present study was testing the external validity (#1) of the YSEX?-15H with self-report measures of the functional aspects of attachment and sexuality. While Personal Goal Attainment as a sexual motive showed negligible (albeit statistically significant) associations with several dimensions of attachment and partner relationship intimacy (i.e., r <|.2|; Ferguson, 2009), more substantial correlations were found for Sexual Hyperactivation, Attachment Avoidance and Anxiety and Relationship as Secondary. These findings suggest that self-focused sexual motivation is related to both secondary strategies of the sexual system, that is, Sexual Hyperactivation and Sexual Deactivation. This is in line with previous findings on the relationship between self-focused sexual motivation and one’s willingness to engage in transactional sex (Birkás et al., 2020; Láng et al., 2021) and on the moderator role of Attachment Avoidance in terms of the impact of self-esteem and intrinsic/extrinsic motivation on one’s openness to transactional sex (Ipolyi et al., 2021). This moderator effect of the Attachment Avoidance realized in different way in the younger and older age groups. In the case of younger respondents, the Attachment Avoidance moderated the effect of self-esteem on the acceptance of sugar relationships: the negative effect of self-esteem decreased to zero with increasing Attachment Avoidance. Furthermore, attachment avoidance moderated the negative effect of intrinsic motivation on the acceptance of sugar relationships (sexual encounter between an older and a younger party in exchange for material benefits), that is, the effect of intrinsic motivation decreased with increasing Attachment Avoidance. For older participants, attachment avoidance moderated the positive effect of extrinsic motivation on the acceptance of sugar relationships, that is, the effect of extrinsic motivation decreased with increasing Attachment Avoidance. These motivational aspects of short-term mating strategies are clearly associated with attachment and sexual system functionality, which may be adequately explained in an evolutionary approach (e.g., Mikulincer & Shaver, 2012; Shaver & Mikulincer, 2006; Simpson & Belsky, 2008).

Relational Reasons as a sexual motive also showed negligible positive correlations with several relationship and attachment dimensions. The lack of larger associations suggests that relationship-focused sexual motivation is related to secure attachment, which is consistent with previous findings showing that the sexual system ceases to be active if and when the primary strategy is successful (e.g., Birnbaum & Reis, 2019; Shaver & Mikulincer, 2006; Szepsenwol et al., 2013). This implies that securely attached partners are likely to enjoy a satisfying sex life in a mutually committed relationship.

Sex as Coping had both negligible and more substantial significant positive correlations with the employed external measures, which suggest that one’s tendency to deal with emotional problems by engaging in sexual activity is related to the secondary strategies of sexual and emotional functioning such as sexual hyperactivation and insecure attachment. These results comport with previous findings obtained with a different methodology, such as the association between hyperactivation and short-term mating interest (Birnbaum et al., 2014), and the association between anxious attachment and commitment to a relationship with negative emotional outcomes, which is due to the congruence of these outcomes with anxiously attached partners’ attachment goals (e.g., Birnbaum, 2018; Davis et al., 2004). Note that the construct measured by the Sex as Coping scale is not identical with either secondary strategy of relationship attachment (Attachment Anxiety and Avoidance) or the secondary functioning of the sexual system (Sexual Hyperactivation or Deactivation). It is more likely, as suggested by the obtained findings, that this type of sexual motivation (Sex as Coping) is related to both alternative secondary strategies, which suggests that the sexual motivation used as a coping strategy may be related to insecure attachment itself.

A final aim of the present study was to test the external validity (#2) of the YSEX?-15H using a measurement of sexual strategies (SOI-R) and a five-factor personality construct (BFI-S). Results showed that the longer version (YSEX?-HSF) and the shorter version (YSEX?-15H) of these questionnaires are similarly related to sociosexuality and the five-factor personality model. Specifically, correlations of acceptable strength (correlation coefficient above 0.2; Ferguson, 2009) show a very similar pattern (same direction, similar in strength) for both instruments for the SOI-R scales. For correlations that are not acceptably strong (correlation coefficients below 0.2; Ferguson, 2009), a slightly different pattern is seen. This implies that unrestricted sociosexuality has a (positive) correlation with only one YSEX? scale (Personal Goal Attainment) and no meaningful strength of association with the others. This correlation holds for both measures. For the five-factor personality model (BFI-S) and sexual motivation (YSEX?), no correlations of acceptable strength (correlation coefficient above 0.2; Ferguson, 2009) were found for either measure. However, the pattern (strength and direction) of correlations between the five-factor personality model and sexual motivation is very similar (in fact, with one exception, identical) for both measures. Overall, the patterns of correlations for both instruments are similar in strength and in the same direction (Meskó et al., 2022). These results confirm provides some evidence for the validity of the new brief instrument for research aimed at measuring sexual motivation.

### Conclusion

The strength of the YSEX?-15H lies in its brevity. It has not been designed to provide comprehensive assessment of the large number of broad and specific sexual motives captured by the long form of the instrument. Nevertheless, it enables researchers to obtain a limited, but manageable scale of major dimensions of sexual motives (in larger samples) and to assess their associations with other psychological functions. It can be optimally used in studies for which time constraints require brief instruments, as part of a large test battery, and in any study that requires either the assessment or control of broad sexual motives. The YEX?-15H may also be useful as an assessment tool in clinical settings where an understanding of client’s sexual motivation may be beneficial for guiding and assessing therapy goals. The YSEX?-15H offers a time-efficient means of assessing one’s fundamental sexual motives.

The instrument has several limitations. First, the self-report methodology used in the present study was based on the assumption that respondents had conscious access to their sexual motives, which is not necessarily true in all cases. Second, data obtained with self-report measures might be distorted by social desirability effects: respondents might suppress socially undesirable responses (e.g., having sex with someone to punish the partner, giving sex for money etc.), whereas they might show a bias toward socially desirable responses (e.g., having sexual contact to express positive emotions toward the partner, being driven by love etc.). Third, although the overall sample used in the study was large and relatively heterogeneous, it was not tested for representativeness. For example, the samples may not have included asexual respondents, who are likely to be uninterested in a study on sexual motivation. Fourth, no data on the participants’ sexual orientation was collected, thus the study enables no conclusions on the associations between sexual orientations and sexual motivation. Exploration of the possible associations is a subject of future research.

## Supplementary Information

Below is the link to the electronic supplementary material.

Supplementary file1 (DOC 37 kb)

Supplementary file2 (DOC 106 kb)

Supplementary file3 (DOC 30 kb)

Supplementary file4 (DOC 31 kb)

## Data Availability

The data that support the findings of this study are available at: https://osf.io/qbjkm/?view_only=fd6b38d0f0b0400e83b0a71e5661e64f.

## Footnotes

1 We transformed the scales in order to conduct parametric tests instead of nonparametric ones and to ensure the comparability of the results across scales. The transformation affects the actual scores; thus, statistical tests aiming to compare means of the scales would not have been possible to be used (but that was not a goal in this study).
