# Supplementary material for: Why Hungarians Have Sex: Development and Validation of a Brief 15-Item Instrument (YSEX?-15H)
Source: Arch Sex Behav. 2022 Aug 8;51(8):4007–22. doi: 10.1007/s10508-022-02380-x (PMC9663389; doi:10.1007/s10508-022-02380-x)
Supplement: Supplementary file 1 — Supplementary file1 (DOC 37 kb) [file 10508_2022_2380_MOESM1_ESM.doc]

Supplement 1. H coefficient and relevant statistical values for each item pair that reached the cut-off of H > 0.5 in the redundancy analysis.

| **Items** | | **H coefficient** |
| --- | --- | --- |
| **49** | 50 | 0.53078 |
| 50 | **51** | 0.55211 |
| 52 | 53 | 0.72874 |
| 52 | **54** | 0.67191 |
| 53 | **54** | 0.70104 |
| **55** | 56 | 0.63891 |
| **55** | 67 | 0.52820 |
| **58** | 59 | 0.67630 |
| **58** | 60 | 0.69159 |
| 59 | 60 | 0.72821 |
| **61** | 62 | 0.69951 |
| **61** | 63 | 0.71942 |
| 62 | 63 | 0.77343 |
| 63 | **70** | 0.49632 |
| **64** | 67 | 0.50466 |
| 68 | **69** | 0.49514 |
| **71** | 72 | 0.76481 |
| **71** | 73 | 0.71701 |
| 72 | 73 | 0.78712 |

*Note*: Retained items are in bold. Please note that some items appeared in more than one redundant pairs, therefore entire pairs were removed in some cases.
