# Supplementary material for: Why Hungarians Have Sex: Development and Validation of a Brief 15-Item Instrument (YSEX?-15H)
Source: Arch Sex Behav. 2022 Aug 8;51(8):4007–22. doi: 10.1007/s10508-022-02380-x (PMC9663389; doi:10.1007/s10508-022-02380-x)
Supplement: Supplementary file 2 — Supplementary file2 (DOC 106 kb) [file 10508_2022_2380_MOESM2_ESM.doc]

Supplement 2. The a parameter values for all items in the item response analysis with 95% confidence intervals (CI), separately for scales.

|  |  |  | **95% CI** | |
| --- | --- | --- | --- | --- |
| **Scale** | **Item** | **a parameter** | **Lower** | **Upper** |
| Personal | 11 | .9577149 | .8814529 | 1.033977 |
| Goal | 5 | 1.00501 | .9163066 | 1.093714 |
| Attainment | 10 | 1.072817 | 1.007904 | 1.137729 |
|  | 7 | 1.170568 | 1.080648 | 1.260488 |
|  | 8 | 1.188097 | 1.082964 | 1.29323 |
|  | 14 | 1.269071 | 1.164765 | 1.373377 |
|  | 12 | 1.319158 | 1.246438 | 1.391879 |
|  | 15 | 1.358502 | 1.246337 | 1.470668 |
|  | 9 | 1.414178 | 1.311536 | 1.51682 |
|  | 21 | 1.449461 | 1.359123 | 1.5398 |
|  | 2 | 1.475539 | 1.401277 | 1.5498 |
|  | 24 | 1.480464 | 1.397547 | 1.56338 |
|  | 3 | 1.481202 | 1.403147 | 1.559258 |
|  | 6 | 1.492923 | 1.381274 | 1.604571 |
|  | 4 | 1.497385 | 1.374796 | 1.619973 |
|  | 13 | 1.576144 | 1.451406 | 1.700881 |
|  | 1 | 1.692765 | 1.613428 | 1.772103 |
|  | 22 | 1.700189 | 1.614859 | 1.785519 |
|  | 23 | 1.705566 | 1.621056 | 1.790075 |
|  | **17** | 1.709202 | 1.628638 | 1.789767 |
|  | **19** | 1.843504 | 1.732039 | 1.954968 |
|  | **16** | 1.848916 | 1.763653 | 1.934179 |
|  | **18** | 1.890214 | 1.802614 | 1.977814 |
|  | **20** | 2.035471 | 1.903382 | 2.16756 |
|  |  |  |  |  |
| Relational | 38 | 1.012541 | .952492 | 1.07259 |
| Reasons | 43 | 1.094396 | 1.021148 | 1.167644 |
|  | 31 | 1.106532 | 1.044689 | 1.168375 |
|  | 28 | 1.124487 | 1.062111 | 1.186863 |
|  | 37 | 1.148148 | 1.077108 | 1.219188 |
|  | 40 | 1.172825 | 1.109623 | 1.236027 |
|  | 25 | 1.210439 | 1.144941 | 1.275937 |
|  | 32 | 1.352172 | 1.2836 | 1.420744 |
|  | 44 | 1.392915 | 1.31402 | 1.471809 |
|  | 35 | 1.412628 | 1.341806 | 1.483451 |
|  | 29 | 1.419784 | 1.348658 | 1.490911 |
|  | 41 | 1.444418 | 1.37447 | 1.514366 |
|  | 34 | 1.460562 | 1.386099 | 1.535025 |
|  | 42 | 1.463991 | 1.391683 | 1.536299 |
|  | 46 | 1.4781 | 1.40576 | 1.55044 |
|  | 26 | 1.480088 | 1.402765 | 1.55741 |
|  | 30 | 1.491286 | 1.417139 | 1.565432 |
|  | 33 | 1.495467 | 1.423208 | 1.567726 |
|  | 45 | 1.514029 | 1.440507 | 1.587551 |
|  | **48** | 1.515803 | 1.441366 | 1.59024 |
|  | **47** | 1.535175 | 1.460633 | 1.609716 |
|  | **27** | 1.577565 | 1.499548 | 1.655582 |
|  | **36** | 1.662356 | 1.58412 | 1.740591 |
|  | **39** | 1.691879 | 1.609208 | 1.77455 |
|  |  |  |  |  |
| Sex | 51 | .6376148 | .5787485 | .6964811 |
| as | 49 | .682814 | .6220311 | .743597 |
| Coping | 54 | 1.241516 | 1.165124 | 1.317908 |
|  | 55 | 1.250032 | 1.169736 | 1.330328 |
|  | 58 | 1.289941 | 1.156719 | 1.423163 |
|  | 57 | 1.437957 | 1.331886 | 1.544029 |
|  | 65 | 1.469406 | 1.379691 | 1.559121 |
|  | 69 | 1.528028 | 1.412319 | 1.643737 |
|  | **66** | 1.535019 | 1.446408 | 1.623631 |
|  | **61** | 1.611808 | 1.517178 | 1.706438 |
|  | **71** | 1.636794 | 1.543306 | 1.730282 |
|  | **70** | 1.677061 | 1.574313 | 1.77981 |
|  | **64** | 1.709818 | 1.590325 | 1.829312 |

*Note*: Retained items are in bold.
