# Supplementary material for: Why Hungarians Have Sex: Development and Validation of a Brief 15-Item Instrument (YSEX?-15H)
Source: Arch Sex Behav. 2022 Aug 8;51(8):4007–22. doi: 10.1007/s10508-022-02380-x (PMC9663389; doi:10.1007/s10508-022-02380-x)
Supplement: Supplementary file 3 — Supplementary file3 (DOC 30 kb) [file 10508_2022_2380_MOESM3_ESM.doc]

Supplement 3. Reasons for Having Sex Questionnaire, Hungarian 15-item Version (YSEX?-15H) in English

YSEX?-15H

People have sex (i.e., sexual intercourse) for many different reasons. Below is a list of some of these reasons. Please indicate how frequently each of the following reasons led you to have sex in the past. For example, if about half of the time you engaged in sexual intercourse you did so because you wanted to celebrate, then you would circle “3” beside question 14. If you have not had sex in the past, use the following scale to indicate what the likelihood that each of the following reasons would lead you to have sex.

I have had sex in the past because...

| 1 | 2 | 3 | 4 | 5 |
| --- | --- | --- | --- | --- |
| None of my sexual experiences | A few of my sexual experiences | Some of my sexual experiences | Many of my sexual experiences | All of my sexual experiences |

1. I wanted to seek experience.

2. I wanted pleasure.

3. I wanted to apologize.

4. It was a seduction/I was seduced.

5. I wanted to recharge myself.

6. I wanted to submit myself.

7. I took the opportunity.

8. Out of passion.

9. I wanted to prove sg to my partner.

10. I wanted to control the other person.

11. I wanted to cheer up the other person.

12. I wanted to comfort the other person.

13. I wanted to demonstrate my power.

14. I wanted to celebrate.

15. I wanted to retain the relationship.

Note:

Personal Goal Attainment: 1, 4, 7, 10, 13.

Relational Reasons: 2, 5, 8, 11, 14.

Sex as Coping: 3, 6, 9, 12, 15.
