# Supplementary material for: Why Hungarians Have Sex: Development and Validation of a Brief 15-Item Instrument (YSEX?-15H)
Source: Arch Sex Behav. 2022 Aug 8;51(8):4007–22. doi: 10.1007/s10508-022-02380-x (PMC9663389; doi:10.1007/s10508-022-02380-x)
Supplement: Supplementary file 4 — Supplementary file4 (DOC 31 kb) [file 10508_2022_2380_MOESM4_ESM.doc]

Supplement 4. Reasons for Having Sex Questionnaire, Hungarian 15-item Version (YSEX?-15H) in Hungarian

Miért szexel ön? kérdőív (YSEX?-15H)

Az ember számos okból létesíthet szexuális kapcsolatot valakivel (pl. közösül). Az alábbiakban egy listát talál majd, amelyen ilyen okok szerepelnek. Kérjük, valamennyi esetben jelölje meg, hogy Önnél milyen gyakran vezettek ezek az okok a múltban ahhoz, hogy valakivel szexeljen. Például, ha egy adott ok egyáltalán nem volt jellemző az ön eddigi szexuális életére akkor jelölje meg a kérdés mellett a "1"-as számot. Ha azonban nagyon gyakran vezetett egy adott ok ahhoz, hogy Ön szexeljen, akkor jelölje meg az "5"-ös számot. Ha nem közösült még életében, akkor azt jelölje a számokkal, hogy milyen okok vezetnék Ön ahhoz, hogy szexeljen (ha ezt tenné).

Azért szexeltem, mert:

| 1 | 2 | 3 | 4 | 5 |
| --- | --- | --- | --- | --- |
| Egyáltalán nincs ilyen szexuális tapasztalatom | Van néhány ilyen szexuális tapasztalatom | Jópár ilyen szexuális tapasztalatom van | Elég sok ilyen szexuális tapasztalatom van | Nagyon sok ilyen szexuális tapasztalatom van |

1. élménykeresés miatt

2. vágytam a gyönyörre

3. bocsánatkérésként

4. csábítás volt

5. feltöltődés miatt

6. behódolás miatt

7. éltem a lehetőséggel

8. szenvedélyből

9. bizonyítás a partnernek

10. uralkodni akartam a másik felett

11. fel akartam vidítani a másikat

12. vigasztalásból

13. demonstrálni akartam a hatalmam

14. ünneplés miatt

15. kapcsolat megtartása miatt

Megjegyzés:

Személyes célok elérése: 1, 4, 7, 10, 13.

Kapcsolati okok: 2, 5, 8, 11, 14.

Szex mint megküzdés: 3, 6, 9, 12, 15.
